# Immunomodulators Inspired by Nature: A Review on Curcumin and Echinacea

**DOI:** 10.3390/molecules23112778

**Published:** 2018-10-26

**Authors:** Michele Catanzaro, Emanuela Corsini, Michela Rosini, Marco Racchi, Cristina Lanni

**Affiliations:** 1 Department of Drug Sciences—Pharmacology Section, University of Pavia, 27100 Pavia, Italy; michele.catanzaro01@universitadipavia.it (M.C.); racchi@unipv.it (M.R.); cristina.lanni@unipv.it (C.L.); 2 Department of Environmental Science and Policy, University of Milano, 20133 Milano, Italy; 3 Department of Pharmacy and Biotechnology, University of Bologna, 40126 Bologna, Italy; michela.rosini@unibo.it

**Keywords:** immune system, immunomodulators, curcumin, curcumin analogues, *Echinacea*, signal transduction pathways

## Abstract

The immune system is an efficient integrated network of cellular elements and chemicals developed to preserve the integrity of the organism against external insults and its correct functioning and balance are essential to avoid the occurrence of a great variety of disorders. To date, evidence from literature highlights an increase in immunological diseases and a great attention has been focused on the development of molecules able to modulate the immune response. There is an enormous global demand for new effective therapies and researchers are investigating new fields. One promising strategy is the use of herbal medicines as integrative, complementary and preventive therapy. The active components in medical plants have always been an important source of clinical therapeutics and the study of their molecular pharmacology is an enormous challenge since they offer a great chemical diversity with often multi-pharmacological activity. In this review, we mainly analysed the immunomodulatory/antinflammatory activity of *Echinacea* spp. and *Curcuma longa*, focusing on some issues of the phytochemical research and on new possible strategies to obtain novel agents to supplement the present therapies.

## 1. Immune System and Immunomodulators

In everyday life, humans are exposed to harmful pathogens and environmental pollutants that can affect the health status and homeostasis of the organism. The immune system (IS) is a complex integrated network of cells, tissues, organs and soluble mediators, evolved to defend the organism against any foreign insult that threatens the integrity of the organism. One of the key features of the IS is its capability to distinguish between the self (own cells and tissues) and the non-self (foreign molecules and microbes of the environment).

The IS involves many types of cells, tissues, and organs. In primary lymphoid organs, bone marrow and thymus, the immune cells are produced and mature; while in the secondary lymphoid organs, lymph nodes, spleen, tonsils and Peyer’s patches in the small intestine, the immune cells circulate and reside during their lifetime. Phagocytic cells, which include monocytes, macrophages and neutrophils, are the most abundant cells of the IS. These cells are capable to engulf and digest pathogens and foreign molecules. Lymphocytes, the second most abundant cells of the IS, are important in the normal immune response to infection and tumors but also in mediating transplant rejection and auto-immunity [1]. They can be distinguished in two different types, called T- and B-cells. All immune cells arise from common hematopoietic stem cells (HSCs) in the bone marrow following haematopoiesis. During the activation of an immune response, lymphocytes exponentially proliferate and differentiate: B-cells turn into plasma cells, a sort of antibody factories that release thousands of antibodies into the bloodstream, whereas T-cells differentiate into different subsets with different specialization [1]. 

The immune response is traditionally classified into *innate* and *adaptive* immunity covering different and specific roles in the immune defence responses. The innate immune system provides an imminent but incomplete defence against a foreign insult and it has not long-term memory [2]. This system includes phagocytic cells, the complement system and various classes of receptors utilized by innate cells, such as toll-like receptors (TLRs). These receptors are a member of patter-recognition receptors family (PPPs) and able to detect conserved pathogens-associated molecular patterns (PAMPs), such as bacterial and fungal cell-wall components (i.e., lipopolysaccharides, bacterial lipopeptides and β-glucans) [3]. Although with some exceptions, TLRs and the other PPPs allow innate cells to discern self from non-self but lack the capacity to discriminate among the non-self-molecules. One exception is represented by TLR5 that seems to be able to respond differently to the flagellins of pathogenic and non-pathogenic bacteria [4]. The adaptive immune response is an antigen-specific system that includes long-lived lymphocytes (memory cells) and their highly specialized receptors [5].

The innate and adaptive systems are not strictly separated but work closely together in a fine-tuning machine. The innate system recognizes the infection and “alerts” the adaptive system through the antigen presentation, that occurs thanks to the major histocompatibility complex (MHC) proteins. The innate cells release also other chemicals signals, such as cytokines and chemokines, to completely activate the adaptive system. Importantly, specialized B and T lymphocytes, known as regulatory cells, manage and stop the immune response once the insult has been counteracted, thus avoiding an excessive response of the IS [6,7].

Despite its high efficiency and specificity, the unbalance of immune responses can be responsible of a plethora of disorders, such as allergy, autoimmune diseases, immunosuppression and AIDS [8,9]. Nowadays, epidemiological data provide evidence of an increase in immunological diseases. This still-growing issue has led to the development of a particular class of molecules, overall called immunomodulators, able to enhance or suppress the immune response in IS-mediated diseases. Whereas immunostimulatory drugs have been developed for their potential applicability to infection, immunodeficiency, and cancer, immunosuppressive drugs are employed to inhibit the immune response in many immune-mediated diseases (i.e., in organ transplantation and autoimmune diseases). Within this context, new and innovative approaches are needed to develop more effective treatments, and nature may represent a source of inspiration. 

## 2. Phytochemical Research

Scientific research on phytochemicals, the active components in medical plants, has always been an important source of clinical therapeutics by offering a great chemical diversity with often multi-pharmacological activity. Since ancient times, phytochemicals have been used in traditional medicine for their properties and health benefits [10]. Many of these natural products have pharmacological or biological activity that can be exploited in pharmaceutical drug discovery and drug design. As an example, polyphenols produced by plants as secondary metabolites are the most abundant antioxidants in the human diet. In the last years, a large number of studies demonstrated the beneficial health effects of their dietary contribution [11,12,13]. Some plant extracts have been proved to modulate the IS response and numerous phytochemicals, included not only polyphenols but also polysaccharides, flavonoids and alkaloids, have been studied for their immunomodulatory activities [14,15,16,17,18]. 

In this review, we focused on the immunomodulatory/antinflammatory activity of Echinacea and turmeric, by analysing some issues of the phytochemical research and, as consequence, new possible strategies to obtain novel agents to supplement the present therapies. 

## 3. *Echinacea* sp.

Echinacea is a genus of nine herbaceous flowering plants in the daisy family (*Asteraceae*; *Compositae*), commonly called coneflowers, originating from eastern and central North America. Echinacea species, parts and preparations have different uses. In particular, three species of Echinacea, namely *E. purpurea*, *E. angustifolia* and *E. pallida*, have been used in Native Americans medicine for centuries as a treatment for respiratory tract infections and inflammatory conditions, including common cold, coughs, bronchitis, and inflammation of mouth and pharynx [19]. Fresh or dry herb, dried rhizome and roots, and alcoholic extracts are commercially available, often combined with ginseng, goldenseal, or garlic [20]. Echinacea preparations belong to the best-selling botanical drugs in the USA and Europe [21].

Inexpensive and effective natural immunomodulators could be of great value in medicine; however, lack of standardization to active ingredients, qualitative and quantitative changes in preparations, lack of rigorous test for efficacy, all contributes to inconsistencies in published results regarding immunomodulatory effects of herbal remedies. Several clinical trials have been carried out with Echinacea preparations and there is evidence of both therapeutic inefficacy and efficacy, depending on preparation and study design. Echinacea can be effective in reducing the duration and severity of cold symptoms, but this effect is noted only with certain preparations of Echinacea, mainly *E. purpurea* [19,22,23]. At this regards, it is interesting the study of Balan et al. [24], which comparing three different *E. purpurea*-based remedies commercially available, namely IMMUNAL drops (succus of *E. purpurea*), IMMUNAL FORTE tablets (*E. purpurea* herbae succus siccum) and ECHINACEA FORTE drops (juice squeezed from fresh flowers of *E. purpurea*), demonstrated important differences in the immunomodulatory effects exerted by the remedies in female Balb/c mice, with stimulation (by IMMUNAL drops and ECHINACEA FORTE), inhibition (by IMMUNAL tablets and ECHINACEA FORTE) and no effects with ECHINACEA FORTE on antibody production or with IMMUNAL drops, depending on the product, highlighting how different preparations can have different modulatory effects.

Echinacea is best known as an immunostimulant, and there are a series of studies that support these immunomodulatory effects, with both increases in innate and specific immunity. However, anti-inflammatory activities are also reported [19,23,25], and anti-viral and anti-microbial effects have also been demonstrated, supporting its use in traditional medicine (see reviews [19,23,25]). This broad spectrum of action indicates that the plant contains in its parts, e.g., leaves, flowers, roots, different active ingredients and that depending on the preparation, e.g., water, alcoholic, oil extracts or dried forms, different compositions are obtained, which can explain its different effects. A thoroughly standardization and testing it is, therefore, critical prior to its use in various immune system malfunctions, as the phytochemical profiles of distinct Echinacea products are highly variable, depending on the harvested plant material, specie used, and extraction protocols.

The folk use of Echinacea is mainly meant to be therapeutic, not prophylactic, as in humans its benefits lie in its ability to shorten the duration and lessen the symptoms of illness, with a post hoc pooling of the available trial results suggesting a relative risk reduction of 10% to 20%, and not in its ability to prevent illness [19,21,26]. Rondanelli et al. [23] also suggested a prophylactic use of highly standardized Echinacea extracts, with a specific phytochemical profile (presence of the polysaccharide Polinacea^TM^, the phenylethanoid echinacoside and substantial lack of alkamides), as a self-care remedy for the prevention of the common cold and to improve the immune response to influence vaccination [23,27]. Authors suggest treatment over 4 months with 2400 mg/day for prophylactic use, and a dose of 4000 mg/day during acute stages of colds, as beneficial for preventing/treating cool [23].

**Figure 1:**
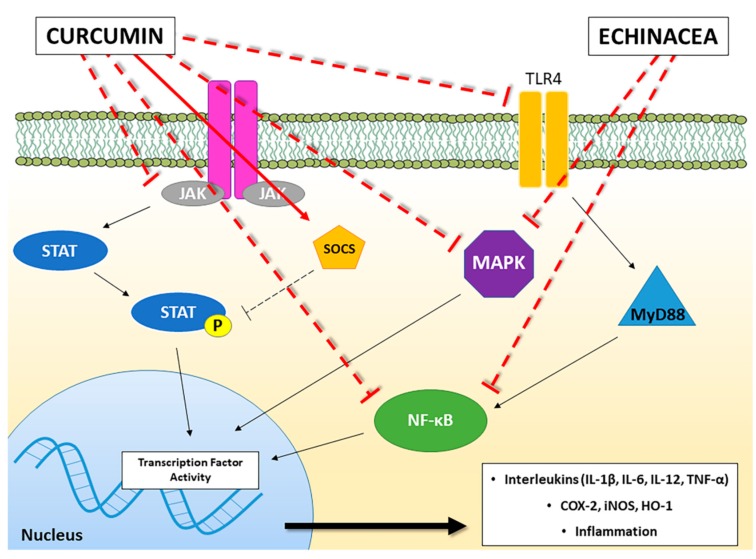
A schematic representation of the main molecular pathways linked to inflammatory and immunomodulatory activities modulated by Curcumin and Echinacea. The solid red line indicates the activation of the pathway, whereas the truncated red line indicates inhibition of the pathway. JAK: Janus kinase; STAT: Signal Transducers and Activators of Transcription; SOCS: Suppressor of Cytokine Signalling proteins; TLR-4: Toll-like Receptor-4; MyD88: Myeloid Differentiation primary response 88; NF-κB: Nuclear Factor kappa B; MAPK: Mitogen-Activated Protein Kinase; COX-2: cyclooxygenase-2; iNOS: inducible Nitric Oxide Synthase; HO-1: Heme Oxygenase-1; IL: Interleukin; TNF: Tumor Necrosis Factor.

Several modulatory effects on immune system have been demonstrated on both innate and acquired immunity (Table 1). Studies suggest that Echinacea stimulates immune functions in both healthy and immune suppressed animals [26]. In macrophages, phagocytosis and cytokine production (increased TNF-α, IL-1, IFN-β) have been enhanced following treatment with Echinacea extracts, increased leukocytes mobility as well as activation of natural killer cells has also been reasonably demonstrated in animals and humans [19,28,29,30]. *E. purpurea* polysaccharide enriched extracts can promote phenotypic and functional maturation of dendritic cells by modulation of JNK, p38 MAPK and NF-κB pathways [31,32] (Figure 1); and can favour M1 macrophage polarization by modulation of JNK pathway [33]. In the study of Wang et al. [32], dendritic cells treated for 24 h with whole plant, stem plus leaf, flower, and root extracts of *E. purpurea* displayed reduced levels of HLA-DR and CD32 expression in a dose-dependent manner compared to the control (untreated) cell samples, with whole plant and stem plus leaf extracts showing the greatest CD32 inhibition compared to the other preparations. These results suggest that whole plant and stem plus leaf extracts have the ability to inhibit dendritic cell maturation. In the study of Fu et al. [33], Echinacea extract (100 µg/mL) significantly activate murine bone-marrow derived macrophage by increasing the expression of CD80, CD86 and MHCII molecules, and by upregulating markers of classically activated macrophages (M1), including CCR7 and the production of IL-1β, IL-6, IL-12p70, TNF-α and NO. In the same study, enhanced phagocytosis and intracellular bactericidal activity were observed [33]. Changes in the numbers and activities of T and B cells have also been described as well as enhanced host resistance, but data are less solid [24,28,30,34,35]. 

Studying the molecular pharmacology of herbal medicines is an enormous challenge due to the fact that herbal extracts are multi-component drugs, working in concert, with multileveled modes of action [36]. Several bioactive phytochemicals have been identified [37]. Among its active ingredients, alkamides (e.g., dodeca-2*E*,4*E*,8*Z*,10*Z*-tetraenoic acid isobutylamide and dodeca-2*E*,4*E*-dienoic acid isobutylamide), polyphenols (e.g., cichoric acid), and polysaccharides can be mentioned. The lipophilic fraction of *E. purpurea* tinctures consists of more than 15 different *N*-alkylamides. *N*-Alkylamide lipids can activate the cannabinoid receptor type 2, with a Ki value of approximately 60 nM, and are supposed to have anti-inflammatory and immunomodulatory activities [38]. Ethanolic extract of *E. purpurea* root and herbal extracts as well as *N*-alkylamide combinations have been shown to produce synergistic pharmacological effects on the endocannabinoid system in vitro, to affect calcium mobilization triggered by PMA, and peroxisome proliferator activated receptor-gamma [38,39,40], and alkylamides from *E. angustifolia* have been demonstrated to inhibit cyclooxygenase and 5-lipoxygenase in vitro [41,42]. In addition, while the expression of the anti-inflammatory cytokine IL-10 was significantly induced in human peripheral blood mononuclear cells, the expression of the pro-inflammatory cytokine TNF-α was inhibited [38,39]. Caffeic acid derivatives, including cichoric acid, caftaric acid, cynarin and chlorogenic acid are believed to be responsible for the wound-healing actions of *E. angustifolia* roots [43]. Besides alkamides and phenolic compounds, the polysaccharide arabinogalactan (75 kDa) from *E. purpurea*, with a structure resembling bacteria lipopolysaccharide, has been identified as the main activator of macrophages [28]. While activating macrophages both in vitro and in vivo, this polysaccharide did not activate B, failed to induce T cells to produce IL-2, IFN-β or IFN-γ, and only caused a slight increase in T-cell proliferation [28]. In Jurkat T-cells, cultured at high density (5 × 10^6^/mL), treated with *E. purpurea* (10–250 μg/mL), containing 80% polysaccharides, predominantly a 10 kDa entity, phenolic compounds, cynarin, cichoric and caftaric acids, but no detectable alkylamides, showed a strong dose-dependent enhancement of production of IL-2 and IFN-γ in response to PMA plus ionomycin was observed [44]. The extract alone had no effect. The high-molecular weight polysaccharides (30–100 kDa) purified by *E. angustifolia* have also been proposed as the anti-inflammatory principles of the plant in mice using the Croton oil ear test [41]. The root oil of *E. angustifolia*, containing 1,8-pentadecadiene, has been reported to inhibit tumor cell growth in mice and rats [45]. 

Different wide-spectrum bioactive components have been identified, which on the one hand indicate that Echinacea extracts have medical potential to be effective for the treatment and prevention of cold and other upper respiratory tract infections and possibly other diseases, while on the other side, the inconsistent results published indicate that effective doses and preparations need to be clearly identified and standardized for a proper therapeutic or prophylactic use. Further studies are required to determine the immunological and pharmacologic potential of Echinacea preparations.

There are still open questions related to long-term use of Echinacea. Although primarily considered for therapeutic purposes, some authors suggest Echinacea for prophylactic use during the winter time [23]. The consequences of Echinacea long-term use (years) are unknown. There were no toxic effects associated with continuous ingestion of different Echinacea preparations for up to 6 months (see review by [46]). Caution with immunostimulants is also warrant, as their use has been associated with development or exacerbation of autoimmunity in genetically predisposed individuals [47,48].

## 4. *Curcuma Longa*

Turmeric (*Curcuma longa*), also known as “Indian saffron” due to its brilliant yellow colour, is a spice herb, member of the ginger family (*Zingiberaceae*) native to the Indian subcontinent and Southeast Asia, having more than a two centuries old scientific history [49]. The worldwide main producer of turmeric is India, which has been used as Ayurvedic remedy and flavouring agent since ancient times (more than 4000 years) [50].

Depending on its origin and growth conditions, turmeric obtained from ground-dried root contains different percentages of volatile and non-volatile oils, proteins, fats, minerals, carbohydrates, curcuminoids and moisture. Commercially available curcumin is a combination of three molecules, together called curcuminoids. Curcumin is the most represented (60–70%), followed by demethoxycurcumin (20–27%) and bisdemethoxycurcumin (10–15%). Curcuminoids differ in potency, efficacy and stability, with no clear supremacy of curcumin over the other two compounds or the whole mixture [51]. Besides curcuminoids, the other active components of turmeric include sesquiterpenes, diterpenes, triterpenoids [52].

To date, many limitations have been recognized for a therapeutic use of curcumin: its poor pharmacokinetic/pharmacodynamic properties, its chemical instability, its low efficacy in different in vitro and in vivo disease models, its toxic profile under certain experimental settings [53] and the very recently suggestion that curcumin may be part of a series of molecules recognized for their interference with biological assays called pan assay interference compounds (PAINS) [54]. Different formulations, changes in the way of administration, the development of nanotechnology-based delivery systems have helped to overcome the critical pharmaceutical issues linked to curcumin pharmacokinetics to improve its therapeutic efficacy and give new hopes for a clinical application of this natural compound [55]. 

Several preclinical and clinical data showed the effectiveness of curcumin in the prevention and treatment of various human diseases including cancer, cardiovascular, inflammatory, metabolic, neurological and skin diseases (reviewed in [56]). Among the different properties referred to curcumin, one of the most studied is the anti-inflammatory profile that may be useful in both acute and chronic inflammation.

The immunomodulatory abilities of curcumin arise from its interaction with various immunomodulators, including not only cellular components, such as dendritic cells, macrophages, and both B and T lymphocytes, but also molecular components involved in the inflammatory processes, such as cytokines and various transcription factors with their downstream signalling pathways [57] (Table 2).

Curcumin has been found to inhibit the immunostimulatory function of dendritic cells (DCs) and to interfere in the myeloid DC maturation. These effects have been related to the suppression of CD80 and CD86 expression, two co-working membrane proteins that provide stimulatory signal necessary for T cell activation, and the impairment in pro-inflammatory cytokine production (IL-12) due to inhibition of MAPK (Mitogen-Activated Protein Kinase) activation and NF-κB (nuclear factor kappa B) translocation [58] (Figure 1). Furthermore, curcumin supplementation in rabbit diet (2, 4 and 6 g/kg) significantly increased serum levels of IgG and IgM, thus suggesting that curcumin can also improve immune functions [59].

The JAK/STAT (Janus kinase/signal transducers and activators of transcription) signalling is a signal transduction pathway directly involved in the cellular homeostasis and in the immune responses, modulating a wide array of cytokines and growth factors involved in cell proliferation, differentiation, cell migration and apoptosis [60]. In vitro concentrations of curcumin ranging from 20 to 50 μM have been reported to inhibit STAT3 phosphorylation in multiple cell types [61,62]. This observation is consistent with data reported by Liu et al. on the capability of curcumin to modulate STAT3 pathway in a mice model with colitis induced by dextran sulfate sodium (DSS) [63]. A significant improvement in the disease activity index and histological injure score compared with control group has been observed following treatment with curcumin (50 mg/kg). Furthermore, also the myeloperoxidase activity (MPO), an index of leukocyte infiltration, and the phosphorylation of STAT3 resulted significantly reduced. Following the decreased DNA-binding activity of STAT3, also the expression of IL-1β and TNF-α were significantly downregulated after treatment with curcumin [63]. More recently, low concentrations (7.5 μM) of curcumin have been found to induce in vitro an anti-inflammatory profile in DCs enhancing the phosphorylation and the activity of STAT3, thus suggesting a biphasic effect of curcumin on STAT3 modulation depending on the range of curcumin concentrations [64]. This observation is quite intriguing and has been also observed when curcumin has been used together with opioids, the drugs of choice for the alleviation of acute and chronic pain and opioid tolerance. In particular, although curcumin seems to be relatively safe to use as a single high dose orally [65], the effect of curcumin on morphine tolerance has been suggested to be biphasic and therefore should be used cautiously [66].

The JAK/STAT signalling pathway is antagonized by Suppressor of Cytokine Signalling proteins (SOCS) that are involved in the regulation of proinflammatory proteins and cytokines production [67]. Guimarães et al. demonstrated that curcumin potently inhibited lipopolysaccharide (LPS)-induced expression of IL-6, TNF-α and prostaglandin-endoperoxide synthase 2 mRNA in murine RAW 264.7 macrophages by preventing the inhibition of SOCS1 and 3 [68]. Curcumin further inhibited LPS-induced p38 MAPK activation by reducing both its phosphorylation and nuclear translocation pointing out the importance of this molecular pathway in inflammatory processes [68] (Figure 1). These data are consistent with the ability of pure curcumin to increase the expression of SOCS1 and SOCS3 proteins in primary myeloproliferative neoplasms cells through suppressing class Ι histone deacetylases (especially HDAC8 activity) [69].

Beside JAK/STAT, another key molecular pathway involved in the inflammation is mediated by NF-κB, a transcription factor regulating the inflammatory response and the immune system homeostasis. NF-κB has been demonstrating to control the expression of inflammatory mediators such as COX-2, inducible nitric oxide synthase (iNOS) and interleukins and to regulate the expression of more than 400 genes involved in inflammation and other chronic diseases [70]. In particular, the modulation of cytokine levels by curcumin has been related to the inhibition of NF-κB signalling pathway [71]. In type 1 diabetes, a T cell-mediated autoimmune disease in which pancreatic β cells are destroyed by the IS, curcumin inhibited pancreatic leucocyte infiltration and preserved insulin-expressing cells [72]. These effects have been related to a reduced NF-κB activation in T cell receptor (TCR)-stimulated NOD lymphocytes and to an impairment of the T cell stimulatory function of dendritic cells, thus leading to reduced secretion of proinflammatory cytokines and nitric oxide (NO) and antigen-presenting cell activity [72]. The involvement of NF-κB and iNOS in anti-inflammatory curcumin effects has also been investigated by Cianciulli et al. [73] in BV-2 murine microglial cells, a specialised population of macrophages found in the central nervous system. Curcumin significantly attenuated the LPS-induced release of NO and pro-inflammatory cytokines, as well as iNOS expression and NF-κB activation [73]. These anti-inflammatory effects have been demonstrated to be mediated by iNOS, COX-2, HO-1, MAPK and NF-κB [74], thus suggesting that curcumin plays an important role in the attenuation of inflammatory responses in the central nervous system by influencing microglial cells through modulation of NF-κB activity. In particular, the induction of NF-κB is dependent on the activation of TLRs. TLR4 is the most studied member of TLRs family and its crucial role in the regulation of immune system response has been well recognized, taking into account that TLR4 receptor agonists have been approved as vaccine adjuvants [75]. The activation of TLR4 recruits MyD88 (myeloid differentiation factor), thus resulting in the induction of NF-κB [76]. The modulation of TLR4/MyD88/NF-κB signalling pathway by curcumin has been demonstrated (Figure 1). Zhu et al. found that curcumin administration in mice following Traumatic Brain Injury (TBI) showed attenuated functional impairment, brain oedema and a reduced neuronal cell death with a general reduction in the activation of microglia/macrophages. In particular, curcumin normalized the LPS-induced upregulation of TLR4, MyD88 and NF-κB both in C57BL/6 mice with an induced TBI in vivo, and in a co-culture system of microglia and neurons, in vitro [77]. Also in rats after spinal cord injury (SCI), curcumin decreased the release of proinflammatory cytokines TNF-α, IL-1β, and IL-6 [78]. Moreover, curcumin down-regulated TLR4 and NF-κB inflammatory signalling pathway, thus ameliorating SCI-induced hind limb locomotion deficits, spinal cord oedema and apoptosis [78]. Similar effects have been observed by Urdzikova et al. in a rat model of SCI, where curcumin, administrated both intraperitoneally and in situ, attenuated glial scar formation by decreasing the levels of Macrophage Inflammatory Protein (MIP1α), IL-2, and Regulated on Activation, Normal T cell Expressed and Secreted (RANTES) production and the NF-κB activity [79]. MIP1α and RANTES are two members of CC chemokine family also known as CCL3 and CCL5 respectively, involved in the inflammatory response and in the recruitment and activation of immune cells. In other different studies, it has been shown that the release of these (and other) chemokines were decreased by curcumin, demonstrating the ability of this compound to modulate the chemotaxis process in the immune response [80,81]. 

The modulatory effects of curcumin on the TLR4/MyD88/NF-κB signalling pathway have been reported not only in brain injury models but also in experimental colitis [82], in LPS-induced mastitis [83] and in *Helicobacter pylori*-induced gastritis [84], pointing out the importance of this pathway in the development of different diseases. 

The anti-inflammatory effects of curcumin have been further used to enhance the efficacy of already approved antimicrobial agents through synergic effects [85]. Bansal et al. demonstrated that curcumin protected BALB/c mice from lung inflammation caused by *Klebsiella pneumoniae* [86]. In this study, mice that received orally curcumin alone or in combination with augmentin showed a significant decrease in neutrophil influx into the lungs and a significant decrease in the production of NO, MPO activity and TNF-α levels [86]. Similar results have been obtained by combining curcumin and clarithromycin [87]. Kim et al. evaluated also the effects of a dietary supplementation with turmeric on systemic and local immune responses on experimental *Eimeria maxima* and *Eimeria tenella* infections in commercial broiler chickens [88]. Dietary supplementation with turmeric enhanced coccidiosis resistance in the chickens with enhanced systemic humoral immunity, as assessed by higher levels of serum antibodies to an *Eimeria* microneme protein, MIC2, and enhanced cellular immunity, as measured by concanavalin A-induced spleen cell proliferation [88]. The antinflammatory effects of curcumin have been tested also against *Mycobacterium tuberculosis* (MTB) infection in an in vitro human macrophage model and have been found to be partially mediated both by NF-κB inhibition and caspase 3 activation [89]. 

Altogether these results underline that curcumin may modulate molecular pathways involved in the inflammation and in the immune response, thus suggesting its putative use as supplement therapy or nutritional approach.

Beyond curcumin, also other bioactive components of *Curcuma longa* have been investigated for their abilities to modulate the immune system. α-turmerone and ar-turmerone, two compounds isolated from the lipophilic fraction *Curcuma longa*, were demonstrated to induce PBMC proliferation and cytokine production [90]. The same effects were stimulated also by the polar fraction of turmeric hot water extracts [91]. Also other curcumin-free turmeric components, such as turmerin, elemene, furanodiene, curdione, bisacurone, cyclocurcumin, calebin A, and germacrone, have been found to exhibit different biological activities including anti-inflammatory and anticancer activity (for a review see [92]). These results suggest the potential use of whole *Curcuma longa* extract to enhance the IS activity in immunosuppressed patients.

## 5. Problematics and Future Perspectives

Nutraceuticals positively influence human health and include a variety of functional foods, fortified foods and dietary supplements (both herbal and not) [93] and their consumption amounts for approximately 20–25% of dietary supplements sales in the USA, pointing out their relevance on the market [94]. Herbal dietary supplements, mainly consisting of herbal extracts, are complex mixtures of phytochemicals which contain not only the principal active compound/s but also minor constituents that can enhance the pharmacological activity of the main active ingredient or lead to adverse effects. The chemical variability and the complexity of the herbal extracts make the study of the pharmacological profile very difficult and this issue is exacerbated whether we consider that different preparations can have different pharmacological effects. One putative hypothesis for batch variability could be ascribed to additional factors that might interfere with the effects of the main active ingredient/s. The inadequate control of quality and standardization of productive processes represent a relevant problem related to the use of dietary supplements. In many studies conducted to determine the effect of natural extracts on immune system, no adequate microbial contamination control protocols have been applied even if it is recognized that microbial endotoxins can modify the parameters and the response of immune system [95]. As regarding the clinical use of curcumin, despite the multi-target activity and its safety at higher doses, one of the major limitations is due to its reduced bioavailability and its low solubility. Several pharmacokinetics studies over the past decades related to absorption, distribution, metabolism and excretion of curcumin have confirmed its poor absorption and rapid metabolism that severely curtails its bioavailability [95]. 

To improve the pharmacokinetic profile of this molecule, alternative strategies have been adopted: new formulations, a change in the way of administration, alternative drug delivery taking advantage from the development of nanotechnology-based delivery systems, such as nanoparticles, liposomes and hydrogels and, finally, the hybridization approach [55,96]. Kumari et al. derivatized curcumin and the lead compound derived, curcumin A, was able to decrease the cell cycle progression of T cells, indicating the anti-inflammatory activities of this new molecule [97]. Jantan et al. tested a series of 43 curcumin diarylpentanoid analogues evaluating their inhibitory effects on the chemotactic activity of phagocytes in vitro, and found that some of them inhibited the migration of human polymorphonuclear leukocytes, suggesting their potential use as chemical leads for the development of new immunomodulatory agents [98]. 

Krishnakumar et al. investigated the bioavailability of a novel formulation of curcumin-impregnated soluble dietary fibre dispersions, which undergoes fermentation in the colon by the action of b-mannanase and may provide protection to curcumin from the degrading enzymes of the upper gastrointestinal tract [99]. This formulation, when orally administered, showed an improved bioavailability when compared to curcuminoids [99,100]. Recently, nanoformulations of curcumin are emerging as a novel substitute to increase aqueous solubility and bioavailability [101]. The entrapment in poly d,l-lactic-*co*-glycolic acid nanoparticles has been demonstrated to be suitable in the transportation of curcumin to target tissues through the epithelia and other biological barriers and in adjuvating its activity increasing an early cell-mediated immune response [102]. Furthermore, curcumin-stabilized silver nanoparticles significantly inhibited the expression of IL-1β, TNF-α, IL-6 and NF-κB in a higher extent than curcumin alone [103]. In addition, lipid nanoparticles encapsulating curcumin were able to prevent metastasis formation and limited the progression of the disease by modulating vascular inflammation in a highly metastatic breast cancer model [104].

Curcumin represents also a starting point for multitarget drug design. Multitarget drugs can be rationally designed by linking, by means of suitable spacers, or fusing the key pharmacophoric functions, or through amalgamation of the pharmacophoric groups essential for activity into one hybrid molecule [105]. Many different curcumin analogues and hybrids have been synthetized and are now under testing phase. The idea to synthesize new hybrids raised by the knowledge that hydroxycinnamoyl recurring motif, present in curcumin, has been shown to modulate several pathways related to aging-related disorders. As an example, Simoni et al. [106,107] synthetized a set of new hybrids, by combining a hydroxycinnamoyl function from curcumin and diallyl sulfides from garlic. This novel design strategy represented an efficient and promising approach, since a catechol derivative with remarkable biological modulating properties has been characterized. This approach could be useful in the near future the development of new efficient molecules to counteract multifactorial diseases.

## 6. Conclusions

The active components in medical plants have always represented an important source of clinical therapeutics since they offer a chemical diversity often associated with a multi-pharmacological activity. Their use in traditional medicine for their properties and health benefits is well recognized since ancient times. Many of these natural products, such as curcumin and Echinacea, have important biological activity that can be exploited in pharmaceutical drug discovery and drug design. However, inconsistencies in published results regarding immunomodulatory effects of herbal remedies have been highlighted, mainly due to limitations such as lack of standardization to active ingredients, qualitative and quantitative changes in preparations and lack of rigorous test for efficacy. There is evidence of both therapeutic inefficacy and efficacy of Echinacea on immune system, depending on preparation and study design. In addition, curcumin show additional limitations related to its poor pharmacokinetic/pharmacodynamic properties, its chemical instability, and its PAINS character [54]. To overcome these critical pharmaceutical issues, new formulations, the direct delivery to the specific tissue taking advantage from the hybridization approach and the development of nanotechnology-based delivery systems have been characterized mainly for curcumin. The use of nanoparticles, in particular, can ensure controlled release of drugs and reduce their toxicity. Noteworthy, natural products might also contain prebiotic components, whose interaction with the host microbiome can significantly impact health and disease. This is a new area of research that would further help optimize the selection of natural products for the maintenance of health and treatment of autoimmune diseases, such as arthritis, systemic lupus and other diseases, and define their mechanisms of action [108]. These approaches may be promising, allowing developing new promising chemical entities, which, however, should be validated through expensive preclinical work to be approved for clinical trials. Within this context, an approach has been recently tried on subjects affected by rheumatoid arthritis, on which a novel, highly bioavailable form of curcumin in a natural turmeric matrix was evaluated for its ability to improve the clinical symptoms of this autoimmune, inflammatory disorder [109]. 

## Figures and Tables

**Table 1:** Main significant immunomodulatory and antinflammatory effects of *Echinacea* in different in vitro studies.

Source	Model & Concentration	Effects	Ref.
In vitro studies			
Arabinogalactan	Isolated mice macrophages; 3.7–500 μg/mL	↑ Macrophages activation ↑ IL-1, TNF-α, IFN-β	[28]
*E. purpurea* extracts	Human Peripheral Blood Mononuclear Cells; ≥0.1 μg/mL	↑ NK function	[29]
*E. purpurea* extracts	Bone Marrow-derived Dendritic Cells; 400 mg/mL	↑ JNK ↑ p38 MAPK, NF-κB	[31]
*E. purpurea* extracts	Human Peripheral Blood Mononuclear Cells; ≥10 μg/mL	↑ DCs differentiation ↓ HLA-DR, CD32	[32]
*E. Purpurea* polysaccharide enriched extract	Bone Marrow-derived Dendritic Cells; 100 μg/mL	↑ Macrophages activation, CCR7 ↑ CD80, CD86, MHCII ↑ IL-1β, IL-6, IL-12p70, TNF-α, NO ↑ Phagocytosis and intracellular bactericidal activity	[33]
Alkylamides from *E. purpurea*	Human whole blood, 5 nM–5 μM	↑ Cannabinoid receptor type 2 ↓ TNF-α,	[38]
Alkylamides from *E. purpurea*	Human Peripheral Blood Mononuclear Cells; 10 μg/mL	↑ Cannabinoid receptor type 2 ↓ TNF-α, ↑ IL-10	[39]
Alkylamides from *E. purpurea*	Jurkat T cells, 330 ng/mL	↑ PPARγ	[40]
*E. Angustifolia* extract	Porcine leukocytes; 50 μM (for its major constituent)	↓ Cyclooxygenase, 5-lipoxygenase	[42]
*E. purpurea* extracts	Jurkat T cells, 10–250 μg/mL	↑ IL-2, IFNγ	[44]

**Table 2:** Main significant immunomodulatory and antinflammatory effects of curcumin in different in vitro and in vivo studies.

Source	Model & Concentration	Effects	Ref.
In vitro studies			
Curcumin	Bone Marrow-derived Dendric Cells; 25 μM	↓ DC maturation ↓ CD80, CD86 ↓ IL-12, MAPK, NF-κB	[57]
Curcumin	Bone Marrow-derived Dendritic Cells; 7.5 μM	↑ STAT3	[63]
Curcumin	Murine macrophage; 10 μM	↓ IL-6, TNF-α, PTGS-2 ↓ p38MAPK ↑ SOCS1, SOCS3	[67]
Curcumin	Myelogenous leukemia cells and human erythroleukemia cells; 20 μM	↑ SOCS1, SOCS3 ↓ HDAC8	[68]
Curcumin	BV-2 microglia cells; ≥10 μM	↓ NF-κB, iNOS ↓ IL-6, TNF-α, IL-1β	[72]
Curcumin	BV-2 microglia cells; ≥10 μM	↓ iNOS, COX-2, HO-1 ↓ MAPK, NF-κB ↓ TNF-α, NO, PGE-2	[73]
Curcumin	Microglial and cortical neurons co-cultures; 2 μM	↓ TLR4, MyD88, NF-κB	[76]
Curcumin	Human promonocytic cells; 30 μM	↓ NF-κB, caspase 3	[88]
α-Turmerone ar-Turmerone	Human Peripheral Blood Mononuclear Cells; 5–10 μg/mL	↑ PBMC proliferation ↑ IL-2, TNF-α	[89]
Polar fraction of turmeric hot water extracts	Human Peripheral Blood Mononuclear Cells; 400 μg/μL	↑ PBMC proliferation ↑ IL-6, TNF-α	[90]
In vivo studies			
Curcumin	Healthy rabbits; 2, 4 and 6 g/kg orally	↑ serum IgG, IgM	[58]
Curcumin	Mice with experimental colitis induced by dextran sulfate sodium (DSS); 50 mg/kg orally	↓ MPO, STAT3 ↓ IL-1β, TNF-α	[62]
Curcumin	Mice with cyclophosphamide (CYP)-induced diabetes; 25 mg/kg intraperitoneally	↓ leucocyte infiltration ↓ NF-κB, NO	[71]
Curcumin	Mice with traumatic brain injury; 100 mg/kg intraperitoneally	↑ activation of microglia/macrophages ↓ TLR4, MyD88, NF-κB	[76]
Curcumin	Rats with traumatic spinal cord injury; 100 mg/kg intraperitoneally	↓ TNF-α, IL-1β, IL-6 ↓ TLR4, NF-κB	[77]
Curcumin	Rats with spinal cord injury; 6 mg/kg intraperitoneally	↓ MIP1α, IL-2, RANTES ↓ NF-κB	[78]
Curcumin	Mice with *K. pneumoniae* induced lung infection; 150 mg/kg orally	↓ leucocyte infiltration ↓ NO, MPO, TNF-α	[85,86]
Curcumin	Broilers with induced *Eimeria maxima* and *Eimeria tenella* infections; 35 mg/kg orally	↑ concanavalin A	[87]
